# Transcriptional recapitulation and subversion of embryonic colon development by mouse colon tumor models and human colon cancer

**DOI:** 10.1186/gb-2007-8-7-r131

**Published:** 2007-07-05

**Authors:** Sergio Kaiser, Young-Kyu Park, Jeffrey L Franklin, Richard B Halberg, Ming Yu, Walter J Jessen, Johannes Freudenberg, Xiaodi Chen, Kevin Haigis, Anil G Jegga, Sue Kong, Bhuvaneswari Sakthivel, Huan Xu, Timothy Reichling, Mohammad Azhar, Gregory P Boivin, Reade B Roberts, Anika C Bissahoyo, Fausto Gonzales, Greg C Bloom, Steven Eschrich, Scott L Carter, Jeremy E Aronow, John Kleimeyer, Michael Kleimeyer, Vivek Ramaswamy, Stephen H Settle, Braden Boone, Shawn Levy, Jonathan M Graff, Thomas Doetschman, Joanna Groden, William F Dove, David W Threadgill, Timothy J Yeatman, Robert J Coffey, Bruce J Aronow

**Affiliations:** 1 Biomedical Informatics, Cincinnati Children's Hospital Medical Center, Cincinnati, OH 45229, USA; 2 Departments of Medicine, and Cell and Developmental Biology, Vanderbilt University and Department of Veterans Affairs Medical Center, Nashville, TN 37232, USA; 3 McArdle Laboratory for Cancer Research, University of Wisconsin, Madison, WI 53706, USA; 4 Department of Genetics and Lineberger Cancer Center, University of North Carolina, Chapel Hill, NC 27599, USA; 5 Molecular Pathology Unit and Center for Cancer Research, Massachusetts General Hospital, Charlestown, MA 02129, USA; 6 Division of Human Cancer Genetics, The Ohio State University College of Medicine, Columbus, Ohio 43210-2207, USA; 7 Institute for Collaborative BioResearch, University of Arizona, Tucson, AZ 85721-0036, USA; 8 University of Cincinnati, Department of Pathology and Laboratory Medicine, Cincinnati, OH 45267, USA; 9 H Lee Moffitt Cancer Center and Research Institute, Tampa, FL 33612, USA; 10 Children's Hospital Informatics Program at the Harvard-MIT Division of Health Sciences and Technology (CHIP@HST), Harvard Medical School, Boston, Massachusetts 02115, USA; 11 University of Texas Southwestern Medical Center at Dallas, Dallas, TX 75390, USA

## Abstract

**Background:**

The expression of carcino-embryonic antigen by colorectal cancer is an example of oncogenic activation of embryonic gene expression. Hypothesizing that oncogenesis-recapitulating-ontogenesis may represent a broad programmatic commitment, we compared gene expression patterns of human colorectal cancers (CRCs) and mouse colon tumor models to those of mouse colon development embryonic days 13.5-18.5.

**Results:**

We report here that 39 colon tumors from four independent mouse models and 100 human CRCs encompassing all clinical stages shared a striking recapitulation of embryonic colon gene expression. Compared to normal adult colon, all mouse and human tumors over-expressed a large cluster of genes highly enriched for functional association to the control of cell cycle progression, proliferation, and migration, including those encoding MYC, AKT2, PLK1 and SPARC. Mouse tumors positive for nuclear β-catenin shifted the shared embryonic pattern to that of early development. Human and mouse tumors differed from normal embryonic colon by their loss of expression modules enriched for tumor suppressors (EDNRB, HSPE, KIT and LSP1). Human CRC adenocarcinomas lost an additional suppressor module (IGFBP4, MAP4K1, PDGFRA, STAB1 and WNT4). Many human tumor samples also gained expression of a coordinately regulated module associated with advanced malignancy (ABCC1, FOXO3A, LIF, PIK3R1, PRNP, TNC, TIMP3 and VEGF).

**Conclusion:**

Cross-species, developmental, and multi-model gene expression patterning comparisons provide an integrated and versatile framework for definition of transcriptional programs associated with oncogenesis. This approach also provides a general method for identifying pattern-specific biomarkers and therapeutic targets. This delineation and categorization of developmental and non-developmental activator and suppressor gene modules can thus facilitate the formulation of sophisticated hypotheses to evaluate potential synergistic effects of targeting within- and between-modules for next-generation combinatorial therapeutics and improved mouse models.

## Abstract 2

Colon tumors from four independent mouse models and 100 human colorectal cancers all exhibited striking recapitulation of embryonic colon gene expression from embryonic days 13.5-18.5.

## Background

The colon is composed of a dynamic and self-renewing epithelium that turns over every three to five days. It is generally accepted that at the base of the crypt, variable numbers (between 1 and 16) of slowly dividing, stationary, pluripotent stem cells give rise to more rapidly proliferating, transient amplifying cells. These cells differentiate chiefly into post-mitotic columnar colonocytes, mucin-secreting goblet cells, and enteroendocrine cells as they migrate from the crypt base to the surface where they are sloughed into the lumen [1]. Several signaling pathways, notably Wnt, Tgfβ, Bmp, Hedgehog and Notch, play pivotal roles in the control of proliferation and differentiation of the developing and adult colon [2]. Their perturbation, via mutation or epigenetic modification, occurs in human colorectal cancer (CRC) and the instillation of these changes via genetic engineering in mice confers a correspondingly high risk for neoplasia in the mouse models. Moreover, tumor cell de-differentiation correlates with key tumor features, such as tumor progression rates, invasiveness, drug resistance and metastatic potential [3-5].

A variety of scientific and organizational obstacles make it a challenging proposition to undertake large-scale comparisons of human cancer to the wide range of genetically engineered mouse models. To evaluate the potential of this approach to provide integrated views of the molecular basis of cancer risk, tumor development and malignant progression, we have undertaken a comparative analysis of a variety of individually developed mouse colon tumor models (reviewed in [6,7]) to human CRC. The *Apc*^*Min*/+ ^(multiple intestinal neoplasia) mouse model harbors a germline mutation in the *Apc *tumor suppressor gene and exhibits multiple tumors in the small intestine and colon [8]. A major function of APC is to regulate the canonical WNT signaling pathway as part of a β-catenin degradation complex. Loss of APC results in a failure to degrade β-catenin, which instead enters the nucleus to act as a transcriptional co-activator with the lymphoid enhancer factor/T-cell factor (LEF/TCF) family of transcription factors [9]. The localization of β-catenin within the nucleus indicates activated canonical WNT signaling. In addition to germline *APC *mutations that occur in persons with familial adenomatous polyposis coli (FAP) and *Apc*^*Min*/+ ^mice, loss of functional APC and activation of canonical WNT signaling occurs in more than 80% of human sporadic CRCs [10]. Similar to the *Apc*^*Min*/+ ^model, tumors in the azoxymethane (AOM) carcinogen model, which occur predominantly in the colon [11], have signaling alterations marked by activated canonical WNT signaling.

Two other mouse models that carry different genetic alterations leading to colon tumor formation are based on the observation that transforming growth factor (TGF)β type II receptor (*TGFBR2*) gene mutations are present in up to 30% of sporadic CRCs and in more than 90% of tumors that occur in patients with the DNA mismatch repair deficiency associated with hereditary non-polyposis colon cancer (HNPCC) [12]. In the mouse, a deficiency of TGFβ1 combined with an absence of T-cells (*Tgfb1*^-/-^*; Rag2*^-/-^) results in a high occurrence of colon cancer [13]. These mice develop adenomas by two months of age, and adenocarcinomas, often mucinous, by three to six months of age. Immunohistochemical analyses of these tumors are negative for nuclear β-catenin, suggesting that TGFβ1 does not suppress tumors via a canonical WNT signaling-dependent pathway. The SMAD family proteins are critical downstream transcription regulators activated by TGFβ signaling, in part through the TGFβ type II receptor. *Smad3*^-/- ^mice also develop intestinal lesions that include colon adenomas and adenocarcinomas by six months of age [14].

To identify transcriptional programs that are significantly activated or repressed in different colon tumor models, we compared gene expression profiles of 100 human CRCs and 39 colonic tumors from the four models of colon cancer to mouse embryonic and mouse and human adult colon. The results of these analyses demonstrate that tumors from the mouse models extensively adopt embryonic gene expression patterns, irrespective of the initiating mutation. Although two of the mouse tumor subtypes were distinguishable by their relative shifts towards early or later stages of embryonic gene expression (driven principally by localization of β-catenin to the nucleus versus the plasma membrane), *Myc *was over-expressed in tumors from all four tumor models. Further, by mapping mouse genes to their corresponding human orthologs, we further show that human CRCs share in the broad over-expression of genes characteristic of colon embryogenesis and the up-regulation of *MYC*, consistent with a fundamental relationship between embryogenesis and tumorigenesis. Large scale similarities could also be found at the level of developmental genes that were not activated in either mouse or human tumors. In addition, there were transcriptional modules consistently activated and repressed in human CRCs that were not found in the mouse models. Taken together, this cross-species, cross-models analytical approach - filtered through the lens of embryonic colon development - provides an integrated view of gene expression patterning that implicates the adoption of a broad program encompassing embryonic activation, developmental arrest, and failed differentiation as a fundamental feature of the biology of human CRC.

## Results

### Strategy for cross-species analysis

**Figure 1:**
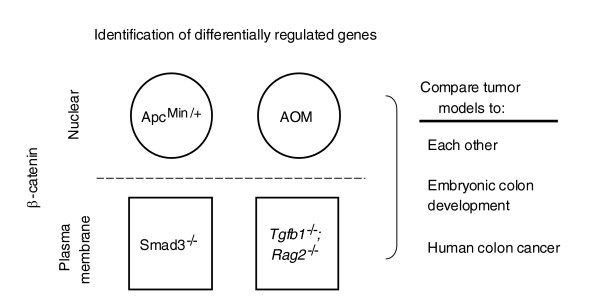
Stratification of murine colon tumor models by localization of β-catenin and plan for analysis. Colon tumors from four etiologically distinct mouse models of CRC were subjected to microarray gene expression profiling. The gene expression profiles from the different mouse model tumors were compared and contrasted to each other, as well as to those from embryonic mouse colon development and 100 human CRCs.

Our strategy for the characterization of mouse models of human CRC (Figure 1) relies on gene expression differences and relative patterning across a range of mouse CRC models, normal mouse colon developmental stages, and human CRCs. Achieving this comparison was facilitated by the use of reference RNAs from whole-mouse and normal adult colon reference RNAs for both mouse and human measurements. Mouse tumor samples were profiled on cDNA microarrays using the embryonic day (E)17.5 whole mouse reference RNA identical to that used previously [15] to examine embryonic mouse colon gene expression dynamics from E13.5 to E18.5, during which time the primitive, undifferentiated, pseudo-stratified colonic endoderm becomes a differentiated, single-layered epithelium. This strategy allowed us to construct a gene expression database of mouse colon tumors in which gene expression levels of the tumors could be referenced, ranked, and statistically compared to an average value among the tumors or to embryonic or adult colon gene expression levels on a per-gene basis. First, we compared the four models with each other, then to mouse colon development, and finally to human CRCs using gene ortholog mapping (Figure 1).

### Mouse colon tumors partition into classes reflecting differential canonical WNT signaling activity

**Figure 2:**
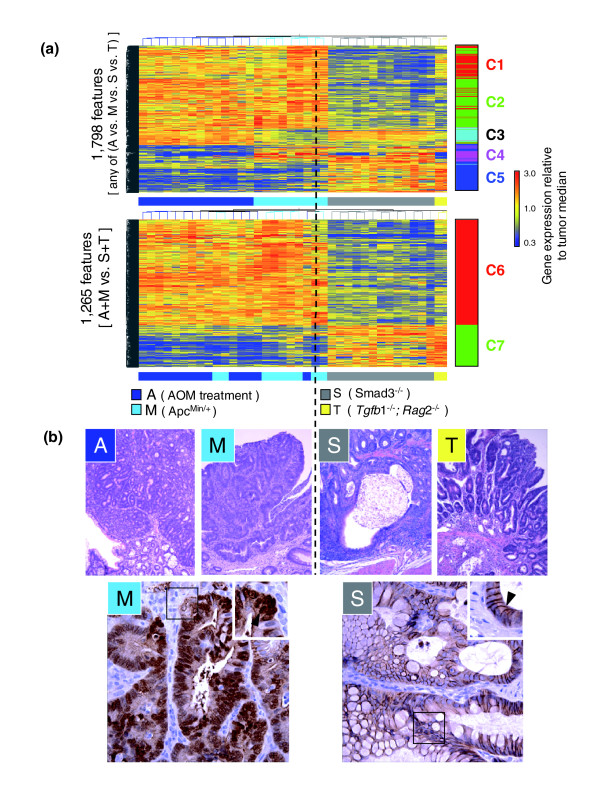
Active canonical WNT signaling (as determined by nuclear β-catenin) stratifies the four murine colon tumor models into two groups. **(a) **Hierarchical clustering of gene transcripts separates the four models into two groups. The upper panel shows 1,798 gene transcripts identified as differentially expressed among any of the four mouse tumor models (Kruskal-Wallis test + Student-Newman-Keuls test + FDR < 5.10^-5^). Results demonstrate that AOM (A) and *Apc*^*Min*/+ ^(M) tumors are transcriptionally more similar to each other than to tumors from *Smad3*^-/- ^(S) and *Tgfb1*^-/-^*; Rag2*^-/- ^(T) mice. Five clusters have been identified (C1-C5) that correspond to the K-means functional clusters listed in Table 1. Please refer to Table 1 for an in-depth description of the functional classification of the genes found in these clusters. The lower panel illustrates the extent of the similarity between A/M and S/T tumors by identifying the top-ranked 1,265 transcripts of the 1,798 that were higher or lower in the two tumor super-groups (rank based on Wilcoxon-Mann-Whitney test for between-group differences with a FDR < 5.10^-5 ^cutoff). Up-regulated transcripts in A/M tumors are highly enriched for genes associated with canonical WNT signaling activity, cell proliferation, chromatin remodeling, cell cycle progression and mitosis; transcripts over-expressed in S/T tumors are highly enriched for genes related to immune and defense responses, endocytosis, transport, oxidoreductase activity, signal transduction and metabolism. **(b) **Representative histologies for each of the four tumor models. The lower panel illustrates the model-dependent localization of β-catenin. Tumors from M (bottom left) and A (not shown) mice exhibited prominent nuclear β-catenin accumulation and reduced cell surface staining. Conversely, tumors from S (bottom right) and T(not shown) mice exhibited retention of plasma membrane β-catenin immunoreactivity. A and M in top panel 100× magnification; S and T 200× magnification. M and S in lower panel both 400× magnification.

To discover gene expression programs underlying differences between etiologically distinct mouse models of CRC, gene expression level values for each transcript in each tumor sample was set to its ratio relative to its median across the series of tumor models. Using non-parametric statistical analyses, 1,798 cDNA transcripts were identified as differentially expressed among the four mouse models of CRC. Five major gene patterns were identified using K-means clustering (clusters C1-C5; Figure 2a, top). Genes belonging to these clusters were strongly associated with annotated gene function categories (see Table 1 for detailed biological descriptions and associations). For example, cluster C1, composed of transcripts that exhibited lower expression in *Smad3*^-/- ^tumors and higher expression in AOM, *Apc*^*Min*/+ ^and *Tgfb1*^-/-^*; Rag2*^-/- ^tumors, contains 391 transcripts, including *Cdk4*, *Ctnnb1*, *Myc*, *Ezh2*, *Mcm2 *and *Tcf3*. Gene list over-representation analysis using Ingenuity Pathway Analysis applications demonstrated highly significant associations to cell cycle progression, replication, post-transcriptional control and cancer. Similarly, cluster C2, composed of 663 transcripts that exhibited high expression in AOM and *Apc*^*Min*/+ ^tumors, but low in *Smad3*^-/- ^and *Tgfb1*^-/-^*; Rag2*^-/- ^tumors, included transcripts for contact growth inhibition (*Metap1*, *Pcyox1*), mitosis (*Mif*, *Pik1*), cell cycle progression and checkpoint control (*Id2*, *Ptp4A2*, *Tp53*).

**Table 1:** Detailed cluster analysis: differential and statistically significant biological functions in clusters C1-C7

Cluster no.	Number of transcripts/ProbeSets (PS)	Reference	Pattern	Biology	Example genes
1	391	Global	Up (A/M/T); down (S)	RNA post-transcriptional modification, cell cycle, DNA replication/recombination/repair, molecular transport, post-translational modification, cellular assembly and organization, cellular movement, cardiovascular system development and function, connective tissue development and function, cancer	Cell cycle progression (Cdk4, Ctnnb1, Id1, Id3, Myc, Pcna, Tcf3), replication of DNA (Idi1, Mcm2, Myc, Orc4l, Pcna, Polb, Set), checkpoint control (Bub3, Myc, Rae1, Smc1l1), invasion of mammary epithelial cells (Ezh2), recovery of ATP (Hspd1, Hspe1), hyperplasia of secretory structure (Cdk4, Ctnnb1, Ptpre, Sdc1), cell proliferation (Id1, Id3, Myc, Pcna)
2	663	Global	Up (A/M); down (S/T)	Cell cycle, cellular response to therapeutics, cellular assembly and organization, molecular transport, connective tissue development and function, genetic disorder, gastrointestinal disease, cancer, Wnt-signaling pathway	Contact growth inhibition of connective tissue cells (Metap2, Pcyox1), mitosis of tumor cells (Mif, Plk1), cell cycle progression (Id2, Tp53), checkpoint control (Mad2l1, Tp53), DNA modification (Apex1, Dnmt3a, Dnmt3b), infiltrating duct carcinoma (Esr1, Ing4), mitosis of tumor cells (Mif, Plk1), myotonic dystrophy (Dmpk, Znf9), Wnt-signaling (Csnk1d, Csnk1e, Lef1, Nlk, Tcf3, Tcf4, Wif1)
3	170	Global	Up (A/S); down (M/T)	Cancer, cell death, cellular development, cellular growth and proliferation, cell cycle	Apoptosis of colon carcinoma cells (Tnfsf10), sarcoma (Ewsr1, Mdm2, Tnfsf10), hyperpoliferation (Map2k7), survival (Mdm2, Nras, Tnfsf10), tumorigenesis (Ewsr1, Mdm2, Nras, Tnfsf10), fibroblast proliferation (Arid5b, E4f1, Map2k7, Mdm2, Nras), mitosis of embryonic cells (E4f1)
4	142	Global	Up (M/S); down (A/T)	Cellular movement, hematological system development and function, immune response, hematological disease, immune and lymphatic system development and function, organ morphology, cell-to-cell signaling and interaction, cell death, molecular transport	Cell movement/chemotaxis (Alox5AP, C3, Ctsb, Cxcl12, Dcn, Fcgr3a, Fgfr1, Hif1a, Igf2, Itgb2, Lsp1, S100A9, Slp1), invasion of tumor cell lines (Cbx5, Ctsb, Cxcl12, Fstl1, Hif1a, Ighg1, Igf2, Itgb2), chemotaxis/migration of leukocytes (C3, Cxcl12, Icam2, Itgb2, Lgals1, Lsp1, S100a9, Slpi), growth of tumor (Fgfr1, Hif1a, Igf2, Igfbp5, Ighg1), invasion of tumor cell lines (Cbx5, Ctsb, Cxcl12, Fstl1, Hif1a, Igf2, Ighg1, Itgb2)
5	432	Global	Up (S/T); down (A/M)	Cell death, neurological disease, drug metabolism, endocrine system development and function, cancer, drug metabolism, lipid metabolism, gastrointestinal disease, organismal functions, organismal injury and abnormalities	Gut epithelium differentiation (Chgb, Klf4, Klf6, Sst), cell death/apoptosis of microglia (Btg1, Casp3, Casp9, Cx3cl1, Grin1, Myd88), uptake of prostaglandin E2 (Slco2a1), tumorigenesis of brain tumor (Nf2, Stat2), tumorigenesis of polyp (Asph, Smad4), aggregatability of colon cancer cell lines (Cd82), cell spreading of colon cancer cell lines (Smad4), contact inhibition of colon cancer cell lines (Prkg1)
6	904	Global	Up (A/M); down (S/T)	Cell proliferation, cell cycle progression and mitosis, DNA replication/recombination/repair, molecular transport, RNA post-transcriptional modification, post-translational modification, cellular growth and proliferation, connective tissue development and function, cancer, gastrointestinal disease, digestive system development and function	Cell cycle progression/proliferation (Cdk4, Clu, Id2, Mki67, Magoh, Myc, Pcna, Tcf3, Tp53), tumor cell mitosis (Mif, Plk1), DNA excision repair (Apex1, Ddb1, Hmgb1, Polb), DNA methylation (Dnmt3a, Dnmt3b), accumulation of colonocytes (Clu, Myc), tumorigenesis (Cd44, Cdk4, Ctnnb1, Esr1, Myc, Prkar1a, Tp53), Wnt-signaling pathway (Csnk1a1, Cskn1d, Cskn1e, Ctnnb1, Lef1, Myc, Nlk, Ppp2cb, Tcf3, Tcf4, Wif1)
7	361	Global	Up (S/T); down (A/M)	Cell death, neurological disease, cancer, drug metabolism, embryonic development, endocrine system development and function, lipid metabolism, organismal injury and abnormalities, infectious disease, immune response, immunological disease, hematological disease; gastrointestinal disease; antigen +presentation pathway	Antigen presentation (B2m, Cd74, H2-D1, HLA-DMA, HLA-DRB, Psmb8, Tap2), embryonic development (C3, Celsr1, Erbb3, Impk, Mcl1), infectious disease (B2m, Ifngr1, Irf1, Myd88, Nr3c1), mast cell chemotaxis (C3, Cx3cl1), apoptosis of microglia (Btg1, Casp3, Cx3cl1, Myd88), tumorigenesis of polyp (Asph, Smad4), transport of prostaglandin E2 (Slco2a1), quantity of colonocytes (Guca2a), gastrointestinal disease (Asph, Cd84, Smad4)

A, AOM-induced; M, *Apc*^*Min*/+^; S, *Smad3*^-/-^; T, *Tgfb1*^-/-^*; Rag2*^-/-^.

From the 1,798 transcripts differentially expressed among the four mouse models of CRC, more than 70% (*n *= 1265) distinguished *Apc*^*Min*/+ ^and AOM tumors versus *Smad3*^-/- ^and *Tgfb1*^-/-^*; Rag2*^-/- ^tumors (Figure 2a, bottom). If a random or equivalent degree of variance occurred among all classes, there would be far less overlap. The majority of this signature (approximately 75%, *n *= 904 features) derived from genes over-expressed in *Apc*^*Min*/+ ^and AOM tumors relative to the *Smad3*^-/- ^and *Tgfb1*^-/-^*; Rag2*^-/- ^tumors (cluster C6). Cluster C6 was functionally enriched for genes linked to canonical WNT signaling (Table 1). These included genes previously identified to be part of this pathway (*Cd44*, *Myc*, *Stra6*, *Tcf1*, *Tcf4 *[16], *Id2*, *Lef1*, *Nkd1*, *Nlk*, *Twist1 *[17], *Catnb*, *Csnk1a1*, *Csnk1d*, *Csnk1e*, *Plat*, *Wif1*) as well as genes that appear to be novel canonical WNT signaling targets (for example, *Cryl1*, *Expi*, *Ifitm3l*, *Pacsin2*, *Sox4 *[16], *Ets2*, *Hnrnpg*, *Hnrpa1*, *Id3*, *Kpnb3*, *Pais*, *Pcna*, *Ranbp11*, *Rbbp4*, *Yes *[18], *Hdac2 *[19]). Moreover, consistent with the over-expression of *Myc *in tumors from the *Apc*^*Min*/+ ^and AOM models, we detected enrichment of *Myc *targets, such as *Apex*, *Eef1d*, *Eif2a*, *Eif4e*, *Hsp90*, *Mif*, *Mitf*, *Npm1 *[20], and the repression of *Nibam *[20].

### Nuclear β-catenin expression distinguishes murine models

**Figure 3:**
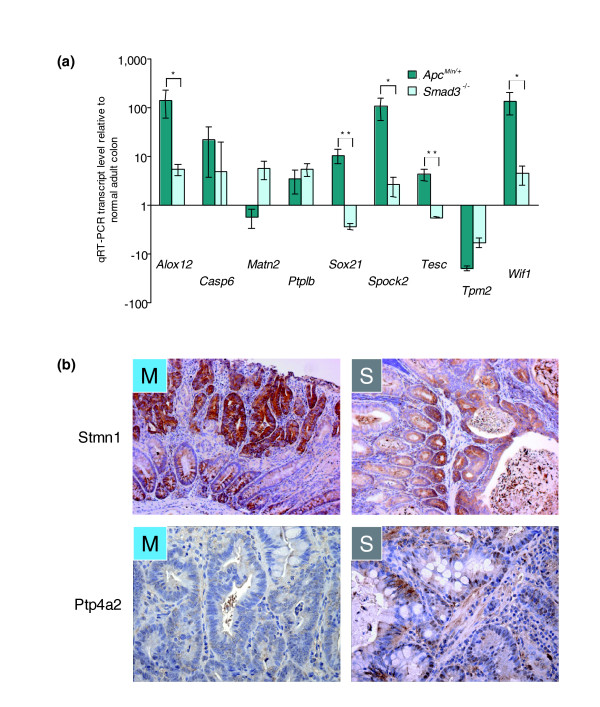
Selective validation of microarray results by qRT-PCR and immunohistochemistry. Differential expression of transcripts identified by the microarray analyses was examined using **(a) **qRT-PCR and **(b) **immunohistochemistry. Additional colon tumors from five *Apc*^*Min*/+ ^(M; nuclear β-catenin-positive) mice and four *Smad3*^-/- ^(S; nuclear β-catenin-negative) mice were harvested, and qRT-PCR was performed on nine genes that exhibited representative strong or subtle patterns in the microarray analyses. All nine patterns detected in the microarray set were validated by the qRT-PCR results. Alox12, Arachidonate 12-lipoxygenase; Casp6, Caspase 6; Matn2, Matrilin 2; Ptplb, Protein tyrosine phosphatase-like B; Sox21, SRY (sex determining region Y)-box 21; Spock2, Sparc/osteonectin, CWCV, and Kazal-like domains proteoglycan (testican) 2; Tesc, Tescalcin; Tpm2, Tropomysin 2; Wif1, WNT inhibitory factor; Stmn1, stathmin 1; Ptp4a2, phosphatase 4a2. In (a), *p < 0.05 and **p < 0.01.

To establish a molecular basis for over-expression of canonical WNT target genes in *Apc*^*Min*/+ ^and AOM tumors, we used immunohistochemistry to characterize the relative cellular distribution of β-catenin. Tumors from *Apc*^*Min*/+ ^(Figure 2b, bottom left panel) and AOM (not shown) mice exhibited strong nuclear β-catenin immunoreactivity and reduced membrane staining (see inset), whereas tumors from *Smad3*^-/- ^(Figure 2b, bottom right panel) and *Tgfb1*^-/-^*; Rag2*^-/- ^(not shown) mice showed strong plasma membrane β-catenin staining with no nuclear accumulation (see inset). Additional tests to confirm the microarray results were also carried out using an independent set of C57BL/6 *Apc*^*Min*/+ ^colon tumor samples analyzed by quantitative real-time PCR (qRT-PCR; Figure 3a) and immunohistochemistry (Figure 3b). All expression patterns identified via microarray analysis were consistent with the qRT-PCR results (*n *= 9 transcripts, chosen for their demonstration of a range of differential expression characteristics). *In situ *hybridization analyses using C57BL/6 *Apc*^*Min*/+ ^colon tumor samples also validated that *Wif*, *Tesc*, *Spock2 *and *Casp6 *were strongly expressed in dysplastic cells of the tumors (data not shown). At the protein level, immunohistochemical analyses confirmed relatively greater expression of the oncoprotein stathmin 1 in *Apc*^*Min*/+ ^mice and tyrosine phosphatase 4a2 in *Smad3*^-/- ^mice (Figure 3b).

Overall, cluster C6 genes (that is, genes with greater up-regulation in tumors from *Apc*^*Min*/+ ^and AOM models than in *Smad3*^-/- ^and *Tgfb1*^-/-^*; Rag2*^-/-^) were consistent with increased tumor cell proliferation (for example, *Myc*, *Pcna*), cytokinesis (for example, *Amot*, *Cxcl5*), chromatin remodeling (for example, *Ets2*, *Hdac2*, *Set*) as well as cell cycle progression and mitosis (for example, *Cdk1*, *Cdk4*, *Cul1*, *Plk1*). It is important to note that *Myc *is up-regulated in all four mouse tumor models relative to normal colon tissue (see below). Biological processes showing increased transcription in tumors from the *Smad3*^-/- ^and *Tgfb1*^-/-^*; Rag2*^-/- ^models (cluster C7) included immune and defense responses (for example, *Il18*, *Irf1*, *Myd88*), endocytosis (for example, *Lrp1*, *Ldlr*, *Rac1*), transport (for example, *Abca3*, *Slc22a5*, *Slc30a4*), and oxidoreductase activity (for example, *Gcdh*, *Prdx6*, *Xdh*) (Table 1). Taken together, these transcriptional observations are both consistent with and extend our understanding of the histological features of the CRC models [7]. For example, while *Apc*^*Min*/+ ^and AOM tumors are characterized by cytologic atypia (that is, nuclear crowding, hyperchromasia, increased nucleus-to-cytoplasm ratios and minimal inflammation), tumors from *Smad3*^-/- ^and *Tgfb1*^-/-^*; Rag2*^-/- ^mice show less overt dysplastic changes but exhibit a significant inflammatory component.

### Large-scale activation of the embryonic colon transcriptome in mouse tumor models

**Figure 4:**
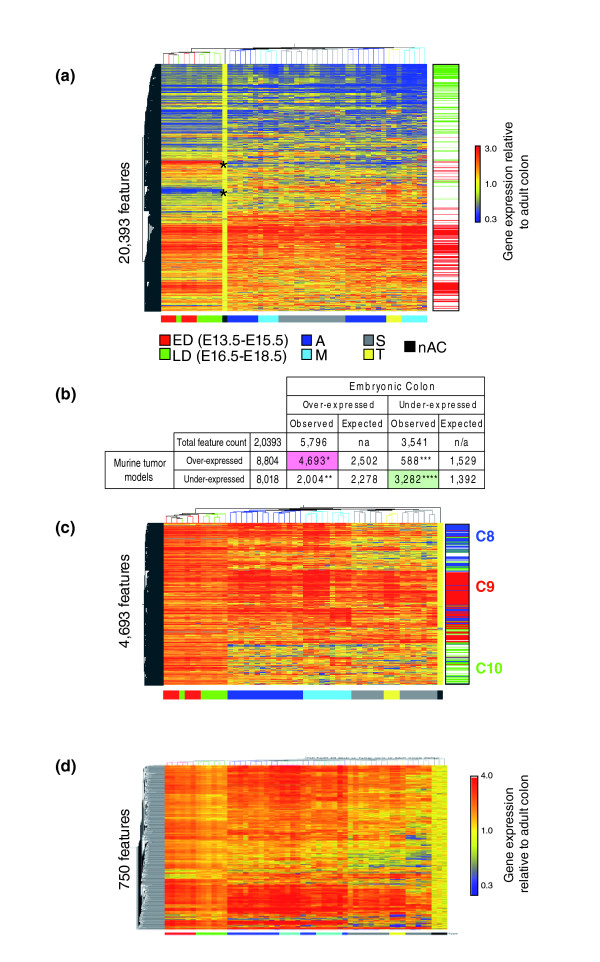
All four murine tumor models exhibit reactivation of embryonic gene expression. The expression level of each gene in each sample was calculated relative to that in adult colon. Genes and samples were subjected to unsupervised hierarchical tree clustering for similarities among genes and tumors. **(a) **Heatmap shows the relative behaviors of 20,393 transcripts that passed basic signal quality filters with gene transcripts shown as separate rows and samples as separate columns. Note that the majority of genes over-expressed in tumors (red) are also over-expressed in embryonic colon; similarly, the genes under-expressed in tumors (blue) are under-expressed in embryonic colon. The color bars to the right indicate the position of 4,693 transcripts over-expressed in both tumors and development (red) or under-expressed in both (green). In addition, there are genes over-expressed in embryonic colon that are under-expressed in tumors and vice versa (asterisks). **(b) **The genes represented in (a) were divided into those over-expressed and under-expressed in embryonic colon and in the tumors, respectively. Fisher's exact test was used to calculate expected overlaps between lists and confirmed significant over-representation of development-regulated signatures among the tumors (*p < 1^-300^, **p < 1.3^-19^, ***p < 4^-296^, ****p < 1^-300^). **(c) **Heatmap showing the behavior of a subset of the transcripts in (a) (*n *= 4,693 features) that were over-expressed in both embryonic colon and tumor samples. Refer to Table 2 for a complete description of the genes associated with these clusters. **(d) **Embryonic gene expression can be further refined into genes expressed differentially during early (ED; E13.5-15.5) and late (LD; E16.5-18.5) embryonic development. Heatmap showing the relative behaviors of 750 transcripts that are highest-ranked for early versus late embryonic regulation. Overall, transcripts with the highest early embryonic expression were expressed at higher levels in nuclear β-catenin-positive tumors (A and M), whereas nuclear β-catenin-negative tumors (S and T) were representative of later stages of embryonic development. Sample groups: ED, early development (E13.5-E15.5); LD, late development (E16.5-E18.5); A, AOM-induced; M, *Apc*^*Min*/+^; T, *Tgfb1*^-/-^*; Rag2*^-/-^; S, *Smad3*^-/-^. Staging: nAC, normal colon. Clusters C8-C10 to the right of the heatmap correspond to the K-means functional clusters listed in Table 2.

We hypothesized that comparisons of genes over-expressed in both colon tumors and embryonic mouse colon could provide valuable insights into tumor programs important for fundamental aspects of tumor growth and regulation of differentiation. To identify genes and observe regulatory patterns that were shared or differed between colon tumors and embryonic development, we applied a global quantitative referencing strategy to both tumor and embryonic samples by calculating the relative expression of each gene as the ratio of its expression in any sample as that relative to its mean level in adult colon. From this adult baseline reference, genes over-expressed in the four mouse tumor models appeared strikingly similar. Moreover, the vast majority of genes over-expressed in tumors were also over-expressed in embryonic colon (Figure 4a). If the fraction of fetal over-expressed genes from the entire microarray (5,796 of 20,393 features; 28.4%) was maintained at a similar occurrence frequency in the tumor over-expressed fraction (8,804 of 20,393), one would expect an overlap of 2,502 transcripts ((8,804/20,393) × 28.4%). Rather, 4,693 out of the 5,796 fetal over-expressed transcripts were observed to be over-expressed in the 8,804 tumor over-expressed genes (Figure 4b). The probability calculated by Fisher's exact test is *p *< 1^-300^, and thus represents highly significant over-representation of fetal genes among the tumor over-expressed genes. Similarly, genes under-expressed in developing colon were disproportionately underexpressed in tumors relative to normal adult colon (3,282 of 3,541; *p *< 1^-300^). Combining these results, approximately 85% of the developmentally regulated transcripts (7,975 out of 9,337 features) were recapitulated in tumor expression patterns relative to adult colon (Figure 4a,b, green and red markers represent the corresponding 7,975 features).

To explore the potential biological significance of genes over-expressed in both embryonic colon development and mouse tumors, we used K-means clustering to generate C8-C10 cluster patterns as shown in a hierarchical tree heatmap (Figure 4c; Table 2). Several sub-patterns were evident, some of which clearly separated *Apc*^*Min*/+ ^and AOM from *Smad3*^-/- ^and *Tgfb1*^-/-^*; Rag2*^-/- ^tumors. One strong cluster, cluster C8, consisted of genes more strongly expressed in *Apc*^*Min*/+ ^and AOM than *Smad3*^-/- ^and *Tgfb1*^-/-^*; Rag2*^-/- ^tumors. This group of genes represented a large fraction of all differences found between nuclear β-catenin-positive (*Apc*^*Min*/+ ^and AOM) and negative (*Smad3*^-/- ^and *Tgfb1*^-/-^*; Rag2*^-/-^) tumors (approximately 45%; 1,636 out of 3,592 features), as well as differences detected between early (that is, E13.5-E15.5, ED) and late (E.16.5-E18.5, LD) embryonic colon developmental stages. Thus, the fraction of developmentally regulated genes that are more characteristic of the earlier stages of normal colon development (E13.5-E15.5), are clearly expressed at higher levels in nuclear β-catenin-positive tumors. This observation is illustrated by 750 transcripts selected solely for stronger expression in ED versus LD (Figure 4d). Note that most of these transcripts overlap with cluster C6 containing 230 features (Figure 2a, lower panel) and illustrate the tendency of the earlier-expressed developmental genes to be more strongly expressed in *Apc*^*Min*/+ ^and AOM mice. In addition, transcripts associated with increased differentiation and maturation, observed at later stages of colon development E16.5-E18.5 (for example, *Klf4 *[21], Crohn's disease-related *Slc22a5/Octn2 *[22], *Slc30a4/Znt4 *[23], *Sst *[24]), were expressed at higher levels by tumors from *Smad3*^-/- ^and *Tgfb1*^-/-^*; Rag2*^-/- ^mice.

**Table 2:** Detailed cluster analysis: differential and statistically significant biological functions in clusters C8-C10

Cluster no.	Number of PS	Reference	Biology	Example genes
8	1,240	Adult	RNA post-transcriptional modification, cell cycle, cellular assembly and organization, DNA replication/recombination/repair, cancer, molecular transport, protein traffic and synthesis, cellular development, gastrointestinal disease, IGF-1 signaling, Wnt-signaling	Mitosis (Ask, Birc5, Bcra1, Cdc2, Cdk4, Chek1, Mad2l1, Mif, Plk1), DNA mismatch repair (Hgmb1, Msh2, Pcna, Rev1l, Xrcc5), cell transformation (Cdc37, Id2, Myc), cell proliferation (Ctnnb1, Pcna, Plat, Plk1, Rala, Top2a), colorectal cancer (Birc5, Brca1, Cdc37, Myc, Top53), IGF-1 signaling (Igf1, Igfb4, Mapk1, Prkc, Ptpn11), Wnt signaling (Csnk1a1, Csnk2a1, Ctnnb1, Gs3kb, Myc, Nlk, Tcf3, Tcf4)
9	1,676	Adult	Protein synthesis, RNA-post transcriptional modification, cancer, connective tissue development and function, embryonic development, organ morphology, tissue morphology, cell-to-cell signaling and interaction, tissue development	Protein synthesis (Csf1, Eif5, Gadd45g, Itgb1, Sars, Tnf, Traf6), transformation (Ccnd1), formation of hepatoma cell line (Hras, Pin1, Shfm1), cell growth (Nrp1, Tnf), invasion of lymphoma cell line (Itgb1, Itgb2), proliferation of ovarian cancer cell lines (Fst, Hras, Itgfb5, Sod2, Sparc), fibroblast cell cycle progression (Ccnf, E2f5, Hras, Map4, Rhoa, Skil), survival of epiblast (Dag1, Itgb1), cell adhesion (Icam1, Itgb1, Itgb2, Lu, Rhoa, Tnf)
10	1,051	Adult	Cell cycle, cellular assembly and organization, DNA replication, recombination/repair, cellular function and maintenance, cancer, cardiovascular system development and function, gene expression, immunological disease, digestive system development and function, activin/inhibin signaling	Cell cycle (Cdk2, Ccnd3, Siah), exocytosis (Nos3, Snap23, Stx6, Vamp2), Burkitt's lymphoma (Dmtf1), cell transformation (Mmp2, Pecam1), angiogenesis (Mdk, Nos3), activation of RNA (Hrsp12, Rps6kb1), development of gastrointestinal tract (Pdgfra, Sptbn1), activin/inhibin signaling (Acvr2b, Bmpr1b, Inha, Map3k7, Mapk8, Tgfbr1)

PS, ProbeSets.

### Human CRCs reactivate an embryonic gene signature

**Figure 5:**
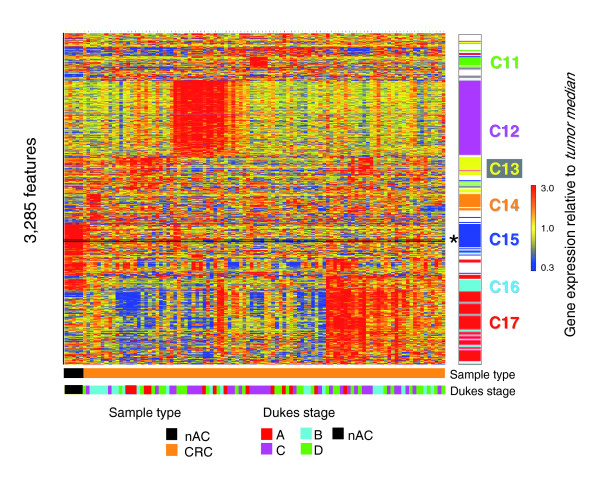
Human CRCs exhibit gene expression profile complexity consistent with significant tumor subclasses. Genes potentially able to distinguish cancer subtypes were identified from Affymetrix HG-U133 plus2 Genechip expression profiles by filtering for 3,285 probe sets that were top-ranked by raw expression and their differential regulation in at least 10 out of 100 human colorectal cancer tumors. Coordinately regulated transcripts and similarly behaving samples were identified via hierarchical tree clustering. Seven different gene clusters (C11-17) were identified that distinguished ten or more tumors from the other tumors. Gene clusters were found to be highly enriched for gene functions listed in Table 3. Data were processed using Robust Microarray Analysis (RMA) with expression value ratios depicted as the relative expression per probe set in each sample relative to the median of its expression across the 100 CRCs. A striking heterogeneity of gene expression was observed, including metallothionein genes in cluster C15 previously shown to be predictive of microsatellite instability (indicated by asterisk), and C17 represented by 734 probesets rich in genes associated with extracellular matrix and connective tissue, tumor invasion and malignancy. Tissue groups: AC, adult colon; CRC, human CRC. Staging: nAC, normal colon; Dukes A-D, human tumors obtained from individuals. Clusters C11-C17 labeled to the right of the heatmap correspond to the K-means functional clusters listed in Table 3.

Since mouse tumors recapitulated developmental signatures irrespective of their etiology, we asked whether a similar commitment to embryonic gene programming was shared by sporadic human CRCs. Tumor classification by microarray profiling is usually accomplished by referencing relative gene expression levels to the median value for each gene across a series of tumor samples. Using this 'between-tumors median normalization' approach, as well as a gene filtering strategy that detects significantly regulated genes in at least 10% of the cases, led to the identification of a set of 3,285 probe sets corresponding to transcripts whose expression was highly varied between independent human tumor cases. As shown in Figure 5, there was striking heterogeneity of gene expression among 100 human CRCs. For example, cluster 15 contained a set of genes (principally metallothionein genes) recently identified to be predictive of microsatellite instability [25,26]. This analysis indicates that human CRCs have a greater level of complexity than the mouse colon tumors studied here (compare Figures 2 and 5). There was no correlation between these distinguishing clusters and the stage of the tumor (note the broad overlapping distributions of Dukes stages A-D across these different clusters). However, as shown in Table 3, gene ontology and network analysis of the individual gene clusters (clusters C11-C17) that were differentially active in subgroups of the tumors, map to genes highly associated with a diverse set of biological functions, including lipid metabolism, digestive tract development and function, immune response and cancer

**Table 3:** Detailed cluster analysis: differential and statistically significant biological functions in clusters C11-C17

Cluster no.	Number of PS	Reference	Biology	Example genes
11	167	Global	Molecular transport, protein traffic, lipid metabolism, small molecule biochemistry, cardiovascular system development, dermatological diseases and conditions, organismal development, organismal injury and abnormalities, cancer, digestive system development and function	Protein excretion (BF, EDNRA, KL), corticosteroid/daunorubicin transport (ABCB1), modification of cholesterol (ABCB1, SULT2B1), neovasculariation of animal (TNFRSF6B, TNFSF11), angiogenesis of granulation tissue (PTGES), blister formation (COL17A1, FRAS1), development of enteroendocrine cells (NEUROD1), crypt size (FOLR1), connective tissue formation (EDNRA, IL7, MSX2, PTGES, WT1), division of mesenchymal cells (BMP7)
12	762	Global	RNA post-translational modification, gene expression, cancer, renal and urological disease, RNA traffic embryonic development, cell-to-cell signaling and interaction, estrogen receptor signaling, EGF signaling, PI3K/AKT signaling	Processing of RNA (HNRPA2B1, HNRPD, HNRPH1, PRPF4B, RBM6, RBPMS, SFPQ, SFRS3, SFRS4, SNRPA1, U2AF1, ZNF638), transactivation of glucocorticoid/thyroid hormone response element (FOXO1A, NCOR1, NR3C1, RORA), tumorigenesis (CD44, CTNNB1, EGFR, NF1, PRKAR1A, PTEN, THBS1), adhesion of tumor cells (CD44, CD47, EGFR, PTK2, THBS1), juvenile/colonic polyposis (CTNBB1, PTEN, SMAD4), IGF1-signalling (CTNBB1, FOXOA1, PTEN, SOS2)
13	213	Global	Cell morphology, cellular development, hematological disease, genetic disorder, embryonic development, cellular assembly and organization, hair and skin development and function, cardiovascular system development and function, cancer, digestive system development and function	Conversion of epithelial cells (ATOH1, DMBT1, FOS), depolarization of cells (CACNA1C, FOS, NTS), development of Goblet/Paneth/enteroendocrine cells (ATOH1), hematological disease (HBA1, HBA2, HBB, GIF), partington syndrome (ARX), muchopolysaccharidosis (HYAL1), Pfeiffer's syndrome (FGFR2), retinoic acid synthesis (ALDH1A1, ALDH1A2), adenoma inflammation (TFF1), density of connective tissue (MIA, TNFRSF11B)
14	161	Global	Cancer, cellular movement, skeletal and muscular disorders, immune response, gastrointestinal disease lipid metabolism, reproductive system disease, small molecule biochemistry, digestive system development and function, tissue development	Migration/invasion of tumor cell lines (CDKN2A, CST6, DPP4, KITLG, LAMA3, LCK, MDK, SERPINB5, TFF2, TGFA), tumorigenesis of intestinal polyp (ASPH), proliferation of tumor cell lines (APRIN, CDKN2A, CST6, IMP3, LITLG, PIWIL1, SLP1, TGFA), cytotoxic reaction (CDKN2A, LCK), invasion of tumor cell lines (CDKN2A, CST6, DPP4, SERPINB5, TFF2, TGFA), tumorigenesis of small intestine (PLA2G4A), size/tumorigenesis of polyp (ASPH, CDKN2A, TGFA)
15	366	Global	Drug metabolism, endocrine system development and function, small molecule biochemistry, lipid metabolism, molecular transport, gene expression, cell death, cell morphology, cancer, gastrointestinal disease, digestive system development and function, tissue development	Steroid metabolism (AKR1C2, CYP3A5, UGT2B15, UGT2B17), conversion of progesterone (AKR1C3, HSD3B2), modification of dopamine (SULT1A3, XDH), oxidation of norepinephrine (MAOA), drug transport (ANCB1, ABCG2), transport of fludarabine (SLC28A2), hydrocortisone uptake (ABCB1), formation of aberrant crypt foci (NR5A2, PTGER4), cell death of enteroendocrine cells (GCG, PYY), growth of crypt cells (NKX2, NKX3)
16	221	Global	Cardiovascular system development and function, cellular movement, hematological system development and function, immune response, cancer, neurological disease, carbohydrate metabolism, organismal development, digestive system development and function, tissue development	Cell movement/proliferation of endothelial cells (ADIPOQ, CXCL12, ENPP2, FGF13, HGF, HHEX, MYH11, PTN), formation of endothelial tube and blood vessel (ADAMTS1, ANGPTL1, CCL11, CXCL12, ENPP2, F13A1, HGF, MEF2C, MYH11, PTEN), cell movement of cancer cells (CXCL12, CD36, HGF, IGF1, L1CAM, SFRP1, PTN), tumorigenesis (AGTR1, CNN1, ENPP2, FGF7, HGF, IGF1, KIT, L1CAM), Hirschprung disease (EDNRB, L1CAM)
17	734	Global	Immune response, cellular movement, hematological system development and function, cell-to-cell signaling and interaction, immune and lymphatic system development and interaction, tissue development, connective tissue disorders, inflammatory disease, cancer	Cell invasion (CD14, CTSB, CTSL, ETS1, FN1, FSCN, FST, INHBA, ITGB2, LOX, MMP2, MMP9, MMP11, MMP12, MMP13, MYLK, OSM, PLAU, RECK, RGS4, RUNX2, S100A4, SPP1, SULF1, TIMP3), adhesion of tumor cells (ADAM12, ANXA1, CCL3, CCL4, FN1, ICAM1, IL6, ITGA4, ITGB2, PLAU, SELE, THBS1), metastasis of carcinoma cell lines (CCL2, DAPK1, S100A4, TWIST1, WISP1), tumor cell spreading (FN1, PLAU, SNAI2, THBS1, TNC), progression of gastric carcinoma (APOE, COL1A1, COL1A2)

PS, ProbeSets.

**Figure 6:**
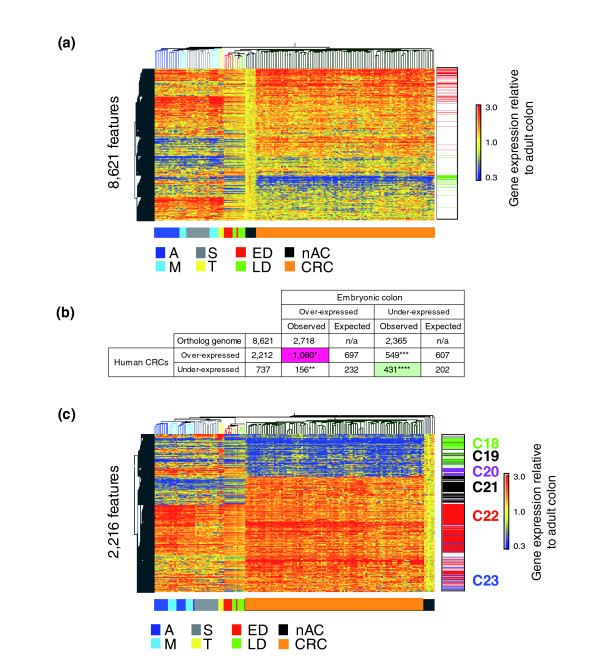
Both human CRCs and mouse colon tumors reactivate an embryonic gene signature. When human and murine tumors are compared, they both broadly re-express an embryonic gene expression pattern. Gene expression profiles from the mouse tumor models and human CRC samples were combined into a single non-redundant gene ortholog genome table structure and subjected to comparative profile analysis. Informative probe-sets from human and mouse platforms were selected, mapped to corresponding ortholog genes, and used to populate a table in which normalized expression for each gene is relative to normal adult colon. **(a) **Heatmap plot for all cross-species gene orthologs both present and successfully measured on both the Affymetrix Hg-U133 and Vanderbilt Mouse NIA 20 K microarrays (*n *= 8,621 features). This representation suggests that a large number of human CRC signatures exhibit similar behaviors in the mouse tumors and during embryonic mouse colon development (sidebar: 1,080 (red) and 431 (green) gene lists from (b)). **(b) **Based on results in (a), four separate gene lists were generated with criteria of over- or under-expression in development or over-expression or under-expression in human CRCs (2,718, 2,365, 2,212, and 737, respectively, with the overlaps shown as a sidebar in (a); red, 1,080 transcripts, and green, 431 transcripts). Genes over-expressed and under-expressed in embryonic mouse colon and human CRCs were found to be over-represented as determined by Fisher's exact test analysis (*p < 7 × 10^-88^, **p < 1 × 10^-76^, ***p < 5 × 10^-4^, ****p < 1 × 10^-76^). **(c) **Heatmap plot of all genes co-regulated in human CRCs and during early (ED) and late (LD) mouse embryonic colon development (*n *= 2,216 features). Six predominant clusters (C18-C23) characterize the transcriptional relationship between human CRC and mouse colon tumor models and embryonic development. Two clusters (C20 and 21) primarily distinguish human CRCs from murine tumors (A, M, S and T). For example, CRC up-regulated transcripts that are either developmentally up- or down-regulated are represented by cluster C22 (*n *= 860 features) and clusters C21/C23 (*n *= 142 features), respectively. Conversely, CRC down-regulated transcripts that are either down- or up-regulated during development are shown in clusters C18/C19 (*n *= 258 features) and cluster C20 (*n *= 42 features), respectively. Interestingly, while approximately 80% and approximately 60% of genes up- and down-regulated in both human CRCs and mouse development were also up- and down-regulated in tumors from the various mouse models, several clusters provide very interesting exceptions: cluster C20 comprises genes down-regulated in human CRCs that are routinely over-expressed in mouse tumors and development; cluster C21 comprises genes robustly expressed in human CRC that are rarely expressed in embryonic colon or murine tumors. Sample groups: ED, early development (E13.5-E15.5); LD, late development (E16.5-E18.5); A, AOM-induced; M, *Apc*^*Min*/+^; T, *Tgfb1*^-/-^*; Rag2*^-/-^; S, *Smad3*^-/-^. Tissue groups: AC, adult colon; CRC, human CRC. Staging: nAC, normal colon.

To evaluate if similar sets of genes are systematically activated or repressed in human CRC, as in the mouse colon tumors, we undertook two procedures to align the data. First, gene expression values for the mouse and human tumors were separately normalized and referenced relative to their respective normal adult colon controls; second, mouse and human gene identifiers were reduced to a single ortholog gene identifier. The latter is a somewhat complex procedure that requires identifying microarray probes from each platform that can be mapped to a single gene ortholog and undertaking a procedure to aggregate redundant probes within a platform (see Materials and methods). This approach allowed the identification of 8,621 gene transcripts on the HG-U133 plus2 and Vanderbilt NIA 20 K cDNA arrays for which relative expression values could be mapped for nearly all mouse and human samples. A clustering-based assessment of expression across the whole mouse-human ortholog gene set identified a large number of transcripts behaving similarly across colon tumors, many irrespective, but some respective of species. Notably, the great majority of genes over-expressed in all tumors were also over-expressed during colon development (Figure 6a). To evaluate the statistical significance of this pattern, we used a Venn overlap filtering strategy and Fisher's exact test analysis. Approximately 50% of the 2,212 ortholog genes over-expressed in at least 10% of the human cancers relative to adult colon were also over-expressed in developing colon. If there was not a selection for developmental genes among those over-expressed in tumors, the expected overlap would be (2,718/8,621) × 2,212 = 697 transcripts. Using Fisher's exact test for the significance of the increased overlap of 1,080 versus 697 transcripts is *p *< 1e-300. Similarly, genes under-expressed in mouse colon development and human CRCs also strongly overlapped (Figure 6b; 431 of 737, *p *< 1e-76). This result is significantly greater than the 8-19% of genes that were estimated to be over-expressed in human colon tumors and fetal gut morphogenesis based upon a computational extrapolation of SAGE data [27]. Thus, our findings not only confirm but also significantly expand and experimentally validate the previously suggested recapitulation of embryonic signatures by human CRCs.

**Figure 7:**
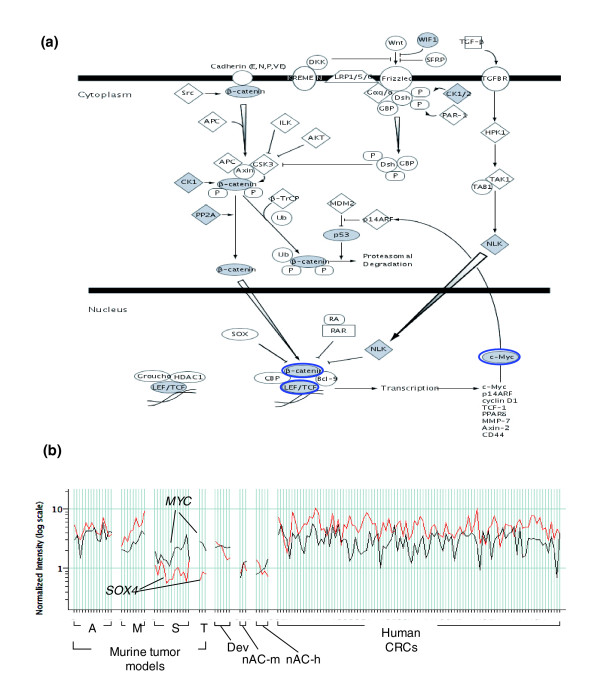
The up-regulated signature in tumors from *Apc*^*Min*/+ ^(M) and AOM (A) models (cluster C6, Figure 2) is enriched with genes associated with the activation of the canonical WNT signaling pathway, as determined by nuclear β-catenin positivity. **(a) **Schematic diagram of the canonical WNT signaling pathway showing elements present in cluster C6 (gene symbols with gray background). Key elements of this pathway (*Ctnnb1*, *Lef1*, *Tcf *and *Myc*) are outlined in blue. **(b**) Relative gene expression for *MYC *and *SOX4 *is plotted for individual murine and human tumors. The relative expression level of *MYC *and *SOX4 *is normalized to adult colon. Note that whereas *Sox4*, a canonical WNT target gene, is expressed at high levels in all human CRCs, A/M tumors and during embryonic mouse colon development, it is not expressed in *Smad3*^-/- ^(S) and *Tgfb1*^-/-^*; Rag2*^-/- ^(T) tumors (black). In contrast, *MYC *is over-expressed in all human and murine tumors and during colonic embryonic development (red), irrespective of the activation of canonical WNT signaling, as determined by nuclear β-catenin positivity (Figure 2). Tissue groups: as above and: nAC-m, normal adult mouse colon; nAC-h, normal adult human colon; Dev, developing mouse colon.

All overlaps between tumor expression and development were pooled to form a set of 2,116 ortholog gene transcripts. This was subjected to hierarchical tree and K-means clustering to define six expression clusters, C18-C23 (Figure 6c; Table 4). These clusters provide an impressive partitioning of groups of genes associated with different biological functions critical for colon development, maturation and oncogenesis. Cluster C22 (860 transcripts of genes strongly expressed both developmentally and across all tumors) is highly enriched with genes associated with cell cycle progression, replication, cancer, tumor morphology and cellular movement. Cluster C18 (258 transcripts down-regulated in mouse and human tumors, as well as in development) is highly enriched in genes associated with digestive tract function, biochemical and lipid metabolism. This cluster is clearly composed of genes associated with the mature GI tract. Thus, as opposed to recapitulating developmental gene activation, the cluster C18 pattern indicates a corresponding arrest of differentiation in both mouse and human tumors. Cluster C23 (142 transcripts over-expressed in all mouse models and human CRC, but with low expression in development) maps to genes highly associated with the disruption of basement membranes, invasion and cell cycle progression, as well as altered transcriptional control. Cluster C21 (313 transcripts in which human tumors somewhat variably express a set of genes that are rarely expressed by the mouse tumors) is remarkable for its composition of genes associated with cell cycle proliferation, tissue disruption and angiogenesis. Thus, while categorically quite similar to cluster C23, the genes in cluster C21 represent a separately regulated module that is enriched for genes associated with invasion. Clusters C21 and C23 reveal sets of genes likely involved in tumor progression. Cluster C22 (with genes over-expressed in all mouse and human tumors and strongly expressed in embryonic colon) represents a group of genes highly correlated with transformation. The top-ranked transcription factor present in this cluster, with regulation independent of β-catenin localization, is *Myc/MYC *(Figure 7b). Although *Myc *was lower in expression in the *Smad3*^-/- ^tumors compared to tumors from the other three models, it was elevated in all four models relative to normal adult colon. *Myc/MYC *was over-expressed in all mouse and human tumors as well as in development. This contrasts with *Sox4*, which is unaltered in expression in the *Smad3*^-/- ^and *Tgfb1*^-/-^*; Rag2*^-/- ^tumors but is up-regulated in AOM and *Apc*^*Min*/+ ^tumors relative to normal adult colon (Figure 7b). *Myc/MYC *over-expression may be independent of nuclear β-catenin status. Increased *Myc/MYC *expression may reflect both activation of canonical Wnt signaling, as it is a target of nuclear β-catenin/TCF [28], and deregulation of TGFβ signaling, as TGFβ1 is known to repress *Myc/MYC *[29-31]. These observations suggest a fundamental role for *Myc/MYC *in colonic neoplasia.

**Table 4:** Detailed cluster analysis: differential and statistically significant biological functions in clusters C18-C23

Cluster no.	Number of PS	Reference	Pattern	Biology	Example genes
18	258	Adult colon	Down (D); down (CRC); down (A/M/S/T)	Lipid metabolism, molecular transport, cell death, cancer, cellular movement, drug metabolism, lipid metabolism, digestive system development and function, small molecule biochemistry, endocrine system development and function, neurological disease	Gut epithelium differentiation (CA4, CA12, CBR1, CHGB, KLF4, KLF9, MCOLN2, SST, TFF3), apoptosis/cell death (CYCS, GSN, KITLG, SST, TFF3, TGFA), cytolisis/crypt damage (ABCB1, KLKR1, PTGER4), formation of aberrant crypt foci (NR5A2, PTGER4), drug transport (ABCB1, ABCG2), migration of tumor cells (EDG2, KITLG, SST, TGFA), quantity of colonocytes (GUCA2A)
19	42	Adult colon	Up (D); down (CRC); down (A/M/S/T)	Digestive system development and function, cancer, small molecule biochemistry, reproductive system development and function, organ morphology	Colon and midgut development (EDNRB), gastrointestinal stromal tumor (KIT), apoptosis of mesothelioma cells (KIT), melanocyte differentiation (EDNRB, KIT), inhibition and morphology of melanoma cells (HSPE, LSP1), adhesion of lymphoma cells (HSPE)
20	91	Adult colon	Up (D); down (CRC); up (A/M/S/T)	Cell death, hematological disease, immunological disease, cell-to-cell signaling and interaction, hematological system development and function, immune response, cancer, cell morphology, tissue development, gastrointestinal disease	Apoptosis of colon carcinoma, cells (BCL2), apoptosis of lymphoma cell lines (BCL2, IGFBP4, MAP4K1, PDGFRA), cell-cell contact of endothelial cells (STAB1), lymphocyte quantity (BCL2, CCR7, CD28, ITGB7, ITK, MUC1, WNT4), proliferation of lymphocytes (CD28, ITK), gastrointestinal stromal tumor (PDGFRA), metastasis (CD28, ENPP2, FKBP1A), transmembrane potential of mitochondria (BCL2, CD28, EYA2, LGALS2, MUC1)
21	313	Adult colon	Down (D); up (CRC); down (A/M/S/T)	Cell death, nervous system development and function, drug metabolism, small molecule biochemistry, cancer, cell cycle, cellular growth and proliferation, tissue development	Melanocyte survival (RB1), proliferation of neuronal progenitor cells (ATM, VEGF), heparin binding (PRNP, TNC, VEGF), dopamine formation (TH), drug resistance (ABCC1), quantity of tumor cell lines (LIF, PIK3R1, RB1, TIMP3, VEGF), transformation (FOXO3A), malignancy of astrocytoma (TNC), tumor vascularization (PTEGS, VEGF), growth of sarcoma cell lines (TIMP3), tissue proliferation (GRP, KRIT1, RB1, RBL2)
22	860	Adult colon	Up (D); up (CRC); up (A/M/S/T)	Cell proliferation, cancer, DNA replication/recombination/repair, cell cycle progression and mitosis, cellular movement, connective tissue development and function, tumor morphology; purine and pyrimidine metabolism, folate metabolism	Cell transformation (Myc), mismatch repair (HMGB1, MSH2, MSH6, PCNA), arrest in mitosis (BIRC5, BUB1B, CDC2.CHEK1, CSE1L, MAD2L1, MIF, PLK1), migration/cytokinesis (ANLN, CDC42, FN1, ITGB5, MSF, SPARC, TOP2A), survival (AKT2, APEX1, BIRC5), gastric carcinoma progression (COL1A1, FUS), folate metabolism (MTHFD1, MTHFD2)
23	142	Adult colon	Down (D); up (CRC); up (A/M/S/T)	Connective tissue development and function, cell-to-cell signaling, development disorder, organismal injury and abnormalities, tumor morphology, hematological system development and function, immune and lymphatic system development and function, cancer	Cell transformation (ESR1, SRC), basal membrane disruption (MMP7), cell extension (ATF3, CD82, IL6, SRC), contact growth inhibition (JUN, IL6), osteocyte differentiation (IL6, JUN, SMAD6, SRC), cell cycle progression (ESR1, IER3, IL6, PSEN2), ERK/MAPK signaling (ESR1, ETS2, PPP1R10, PPP2R5C), development of tumor (CXCL6, ESR1, IER3, IL6, JUN), invasion of colon cancer cell lines (CD82, SRC), colon cancer (JUN, PDGFRL, SRC)

A, AOM-induced; M, *Apc*^*Min*/+^; PS, ProbeSets; S, *Smad3*^-/-^; T, *Tgfb1*^-/-^*; Rag2*^-/-^.

## Discussion

Numerous mouse models of intestinal neoplasia have been developed, each with unique characteristics. The models constructed to date, however, do not fully represent the complexity of human CRCs principally because most are unigenic in origin and produce primarily adenomas and early stage cancers. Although models like *Apc*^*Min*/+ ^show molecular similarities to human CRCs, such as initiation of adenoma formation by inactivation of *Apc*, little is known about the molecular similarities of tumors from the different mouse models. It is also unknown how such common and perhaps large-scale molecular changes in mouse models relate to the molecular programming of human CRC. To shed light on the underlying molecular changes in tumors from mouse models and human CRC, we assessed the relationship at the molecular level of four widely used, but genetically distinct, mouse models that develop colon tumors. A subsequent analysis of the models in the context of embryonic mouse colon development was also undertaken. Finally, to identify consensus species-independent cancer signatures that may define gene expression changes common to all CRCs, we projected relevant mouse model signatures onto a large set of human primary CRCs of varied histopathology and stage.

### Differential canonical WNT signaling activity discriminates two major classes of mouse models of CRC with distinct molecular characteristics

Tumors from mouse models of CRC exhibit significant phenotypic diversity [6], and, therefore, were expected to exhibit differential gene expression patterns. Using a combination of inter-model and normal adult gene expression level referencing, our analysis of tumors from mouse models of CRC has revealed a low complexity between models and strains, and has identified common and unique transcriptional patterns associated with a variety of biological processes and pathway-associated activities. Our results demonstrate an imbalance between proliferation and differentiation, with nuclear β-catenin-positive tumors being more proliferative, less differentiated and with lower immunogenic characteristics than tumors from nuclear β-catenin-negative tumors. Mouse tumors characterized by signatures of relative up-regulation of genes associated with cell cycle progression also showed increased canonical WNT signaling activity (*Apc*^*Min*/+ ^and AOM). Tumors from mouse models not showing canonical WNT signaling pathway activation (*Smad3*^-/- ^and *Tgfb1*^-/-^*; Rag2*^-/-^) were characterized by up-regulation of genes associated with inflammatory and innate immunological responses, and intestinal epithelial cell differentiation. Recent studies have indicated that chronic inflammation caused either by infection with *Helicobacter pylori *[32] or *Helicobacter hepaticus *[13] is a prerequisite for intestinal tumor development in *Smad3*^-/- ^and *Tgfb1*^-/-^*; Rag2*^-/- ^mice, respectively.

The activation of canonical WNT signaling in AOM tumors was identified using a between-tumor global median normalization to gene expression data. However, when tumor sample expression was referenced to that of normal adult intestinal tissue, many more genes are up-regulated, including developmental genes that are not dependent on nuclear β-catenin. That canonical WNT signaling-related genes are altered similarly in both AOM and *Apc*^*Min*/+ ^tumors suggests biological similarities between the two models. In addition, the relatively consistent programming within the AOM model also emphasizes its value for examining the more complicated genetics that result in strain-specific sensitivity to environmental agents that induce cancer.

Activation of canonical WNT signaling leads to nuclear translocation of β-catenin and, through its interaction with LEF/TCF, the regulation of genes relevant to embryonic development and proliferation [16], as well as stem cell self-renewal [33]. Consequently, the activated canonical WNT signaling observed in *Apc*^*Min*/+ ^and AOM models suggests that tumors may arise as a consequence of proliferation of the stem cell or 'transient amplifying' compartment. In the colonic crypt, loss of TCF4 [34] or DKK1 over-expression [35] promotes loss of stem cells, suggesting that canonical WNT signaling is required for the maintenance of the intestinal stem cell compartment [34-36]. Conversely, increased nuclear β-catenin/TCF4 activity imposes a crypt progenitor phenotype on tumor cells [18]. In this study, we identified transcriptional activation of the canonical WNT signaling pathway in tumors from *Apc*^*Min*/+ ^and AOM mice. This was confirmed by immunohistochemistry (Figure 2b).

In colon tumors and perhaps intestinal stem cells, activation of canonical WNT signaling promotes a hyperproliferative state. Proliferation-related characteristics of nuclear β-catenin-positive tumors include increased expression of *CCND1*, *MYC*, *PCNA *[18], and *Sox*4 [16]. These genes were also identified as a component of our nuclear-β-catenin-positive signatures. In turn, increased MYC decreases intestinal cell differentiation by binding to and repressing the *Cdkn1a *(coding for p21^CIP1/WAF1^) promoter [37], the gene encoding Wnt-inhibitory factor Wif1, the gene encoding the negative regulator of WNT Naked1 [38], and the gene encoding the Tak1/Nemo-like kinase, Nlk [39]. *Wif1 *displays a graded expression in colonic tissue, with higher expression in the stem cell compartments and lower expression in the more differentiated cells at the luminal surface, suggesting that *Wif1 *may contribute to stem cell pool maintenance independent of WNT signaling inhibition. [40].

Canonical WNT signaling not only governs intestinal cell proliferation, but also cell differentiation and cell positioning along the crypt-lumen axis of epithelial differentiation. Increased canonical WNT signaling activity enhances MATH1-mediated amplification of the gut secretory lineages [41]. Canonical WNT signaling also influences cell positioning by regulating the gradient of EPHB2/EPHB3 and EPHB1 ligand expression [42,43]. Together, our data suggest a complex imbalance of crypt homeostasis due to enhanced canonical WNT activity.

Our results indicate that tumors arising in response to abnormal TGFβ1/SMAD signaling [14,44] are similar to one another in their specific gene signatures and broadly distinct from those with activated canonical WNT signaling by their absence of nuclear β-catenin. Unique to the dysregulated TGFβ1/SMAD4 signaling models is the strong signature of an immunologically altered state, with up-regulation of genes determining immune and defense responses, such as *Il18*, *Irf1 *and mucin pathway-associated genes. Again, these tumors are usually characterized by a strong inflammatory component when evaluated histopathologically, even in the absence of T- and B-cells such as in the *Tgfb1*^-/-^*; Rag2*^-/- ^background.

As shown in Figure 2a, the microarray patterns of gene expression for AOM and *Apc*^*Min*/+ ^tumors are mirror images of those for *Tgfb1*^-/-^*; Rag2*^-/- ^tumors. It is perhaps not surprising that combining these two transcriptional programs results in increased number and invasiveness of colonic tumors as recently reported for *Apc*^*Min*/+ ^mice crossed to *Smad3*^-/- ^mice [45]. Moreover, combined activation of canonical WNT signaling and inhibition of TGFβ signaling also results in more advanced intestinal tumors in *Apc*^*delta*716/+^*; Smad4*^+/- ^mice [46], and intestine-specific deletion of the type II TGFβ receptor in *Apc*^1638*N*/*wt *^mice [47].

The findings that shared over-expressed signatures are identifiable in all four mouse models of CRC, which are also representative of the majority of embryonic colonic over-expressed signatures, and that these signatures are also present in all human CRCs, suggest that colon tumors may arise independently of canonical WNT signaling status. A likely candidate to impart this oncogenic signaling is *Myc*, which is an embryonic up-regulated transcript that is also upregulated in all human CRCs and mouse tumor models independently of nuclear β-catenin status.

### Embryology provides insight into the biology of mouse and human colon tumors

It has long been suggested that cancer represents a reversion to an embryonic state, partly based upon the observation that several oncofetal antigens are diagnostic for some tumors [48,49]. To assess the embryology-related aspects of tumorigenesis and tumor progression in CRC, we analyzed and compared the transcriptomes of normal mouse colon development and models of CRC. Our data show that developmentally regulated genes represent approximately 56% of mouse tumor signatures, and that the tumor signatures from the four mouse models recapitulate approximately 85% of developmentally regulated genes.

**Figure 8:**
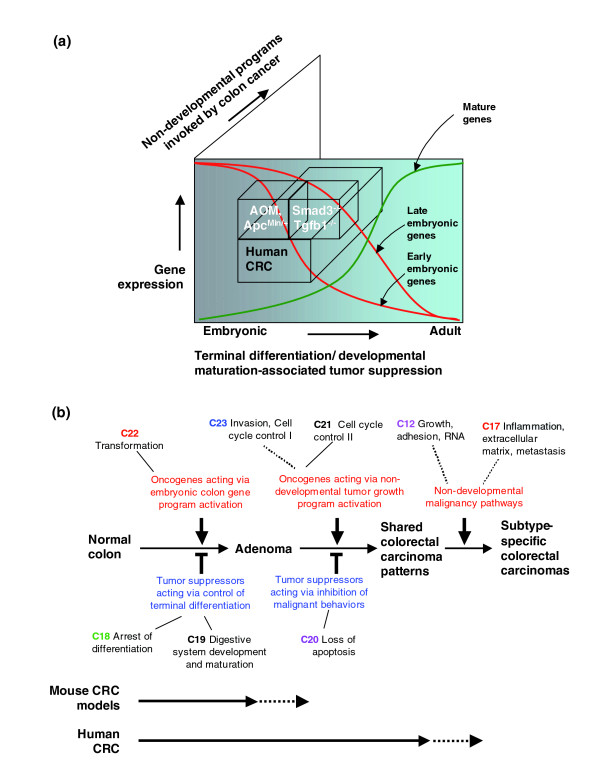
An integrated view of colon cancer transcriptional programs provides novel insight into neoplasia. Murine colon tumor adenomas and human CRCs both show adoption and dysregulation of signatures tightly controlled during embryonic mouse colon development. The use of etiologically distinct mouse models of colon cancer allows for the identification of models that resemble different stages of embryonic mouse colon development and that are recapitulated by specific tumor types. **(a) **All tumors exhibit large-scale activation of developmental patterns. Nuclear β-catenin-positive (*Apc*^*Min*/+ ^and AOM) tumors map more strongly to early development stages during (more proliferative, less differentiated), whereas nuclear β-catenin-negative (*Tgfb1*^-/-^*; Rag2*^-/- ^and *Smad3*^-/-^) tumors map more strongly to later stages consistent with increased epithelial differentiation. **(b) **Overall representation of the relationship of mouse colon tumor models and human CRC to development and non-developmental expression patterns. Gene expression clusters mapped to the progression of adenomatous and carcinomatous transformation identified in Figures 5 and 6 are shown as the clusters of genes whose expression is either gained or lost associated with the stage of progression. For example normal development could be considered as 'subverted' if there is an absence of expression of genes normally expressed at high level in the developing colon that fail to be expressed in tumors (for example, C18, C19), or that are activated in tumor but not normally expressed in development (C20). Upregulated clusters are enriched for genes with known oncogenic functions and down-regulated clusters for genes associated with tumor suppression. Both mouse colon tumor models and human CRC share in the activation of embryonic colon expression (C22), or partially overlap (C23, dotted lines) the loss or repression of adult differentiation-associated genes (C19), and the loss of tumor suppressor genes (C18). Many human CRCs also lack the expression of additional tumor suppressor programs and gain the expression of oncogenes that are not over-expressed during normal developmental morphogenesis (C21).

There are at least two regulatory programs that determine the expression of developmental genes by mouse tumors (Figures 2, 4, and 8). The simpler program is evident by the over-expression of the earliest genes of colon development by the nuclear β-catenin-positive models. The more subtle program could be detected only in reference to adult colon and is highly shared by nuclear β-catenin-negative models. This program, though modified by nuclear β-catenin status, is represented by a large scale over-expression of developmentally expressed genes in tumors that are both positive and negative for canonical WNT signaling. Genes found within this signature have a large overlap with those present in the colon at later developmental stages (E16.5-E18.5).

How do genes tightly regulated during mouse colon development become activated in colon tumors? While activated canonical WNT signaling imparts a strong influence, its absence in *Tgfb1*^-/-^*; Rag2*^-/- ^and *Smad3*^-/- ^tumors, as determined by the absence of nuclear β-catenin, did not prevent the large scale activation of developmental/embryonic gene expression. One mechanism may be through epigenetic alterations. In human CRCs, these types of alterations in gene expression programs [50] suggest a link between cellular homeostasis and tumorigenesis. The recruitment of histone acetyltransferases and histone deacetylases (HDACs) are key steps in the regulation of cell proliferation and differentiation during normal development and carcinogenesis [51]. Induction of *Hdac2 *expression occurs in 82% of human CRCs as well as in tumors from *Apc*^*Min*/+ ^mice [19]. Alternatively, common regulatory controls may operate in parallel growth and differentiation/anti-diifferentiation pathways such that a single or small subset of regulators, such as MYC or one or more micro RNAs, may be responsible for the control of multiple pathways. Indeed, consistent with our observation of nuclear β-catenin-independent activation of *Myc *in all mouse models and across the board for human CRC, deletion of *Myc *has recently been demonstrated to completely abrogate nuclear β-catenin-driven small bowel oncogenesis in mouse models [52].

### Comparative analysis reveals underlying development-related signatures in human CRCs

As shown in Figure 5, considerable and intriguing heterogeneity of human CRC is observed among genes highly relevant for differential malignant behavior. However, employing between-tumors normalization and referencing strategies prevents the detection of gene expression patterns that are shared between tumors. Using the adult normal colon as a reference, as shown in Figure 6, a large fraction of differential gene expression relative to adult colon could be demonstrated that recapitulated developmental gene expression by virtue of both activating embryonic colon gene expression and failing to express genes associated with normal colon maturation. Within these developmentally regulated gene sets, our analyses revealed little evidence of CRC subsets, including those suggestive of nuclear β-catenin negative tumors that might approximate the *Smad3*^-/- ^and *Tgfb1*^-/-^*; Rag2*^-/- ^signature. Our inability to identify distinct subclasses with respect to developmental genes in the human CRCs is perhaps not surprising in that over 80% of microsatellite-unstable (MSI+) CRCs from HNPCC families exhibit nuclear β-catenin [53]. In addition, within the developmental genes, little evidence was apparent for signatures related to MSI+ tumors, often associated with HNPCC, although some of this type of signature was perhaps apparent in the median normalized depiction of the tumors as highlighted in Figure 5.

This report constitutes a comprehensive molecular evaluation and comparison of mouse and human colon tumor gene expression profiles. We have greatly improved our ability to compare tumor gene expression profiles between mouse and human tumors by using a referencing strategy in which gene expression levels in the tumor samples are analyzed in relation to gene expression in corresponding normal colon epithelium. This approach has revealed that gene expression patterns are both shared and distinct between mouse models and human CRCs. Although several recent studies have suggested that tumors recapitulate embryonic gene expression [16,27,54,55], the present study demonstrates the magnitude of this similarity.

Finally, our results suggest that comparisons made between mouse tumor models, developing embryonic tissues, and human CRCs provides a powerful biological framework from which to observe shared and unique genetic programs associated with human cancer. While ortholog-gene based analyses have been used previously to obtain direct comparison of the molecular features of mouse and human hepatocellular carcinomas [56], our results provide striking support for the hypothesis that cancer represents a subversion of normal embryonic development. By inclusion of detailed mouse embryonic and developmental profile information, our results have revealed critical similarities and differences between the mouse and human tumors that are particularly revealing of oncogenic and tumor suppressor programs, some genes from which should be useful for development of diagnostic biomarkers and identification of therapeutic targets and pathways.

## Materials and methods

### Mouse models, human CRC patients and tumor collection

#### Mouse tumors

All tumors were isolated as spontaneously occurring lesions in *Apc*^*Min*/+ ^[57], *Smad3*^-/- ^[58], and *Tgfb1*^-/-^*; Rag2*^-/-^, collected at three-to-nine months of age depending on the model (for a review, see [6]). The only exceptions were two *Apc*^*Min*/+ ^tumors, UW_3_2778 and UW_6_2748, that were 13 and 14 months and the three *Tgfb1*^-/-^*; Rag2*^-/- ^tumors, all five of which had histological features of locally invasive carcinoma [7]. Three- to four-month old mice from various AXB recombinant inbred lines were treated with AOM doses chosen for enhancement of inter-strain differences in susceptibility [11]. Mice were given four weekly i.p. injections of 10 mg AOM per kg body weight, and tumors were collected six months after the first injection. Animals were euthanized with CO_2_, colons removed, flushed with 1× phosphate-buffered saline (PBS), and laid out on Whatman 3 MM paper. A summary of the mouse strains, mutant alleles and source laboratories is presented in Table 5. All tumors were obtained from the colon only, the particular segment of which is indicated in the Gene Expression Omnibus (GEO) database [59] reposited sample information (GSE5261). The majority of *Tgfb1*^-/-^*; Rag2*^-/- ^and *Smad3*^-/- ^tumors occur in the cecum and proximal colon and all samples isolated for characterization were obtained from there. In contrast, tumors isolated from *Apc*^*Min*/+ ^and AOM mice occurred predominantly in the mid- and distal colon. A small portion of the tumor was placed in formalin for histology, with the remainder finely dissected into RNAlater (Ambion Inc., Austin, TX, USA) and stored at -20°C. Normal adult colon RNA for reference was obtained from whole colon samples harvested from ten eight-week-old C57BL/6 male mice. The tissue was lysed in Trizol Reagent (Invitrogen Systems Inc., Carlsbad, CA, USA) and homogenized. Total RNA was purified using a Qiagen kit (USA-Qiagen Inc., Valencia, CA, USA).

**Table 5:** Mouse models of colon cancer

Model	*Mm *strain	N	Tumor-generating laboratory
Azoxymethane (AOM)	A × B	14	Threadgill
*Apc*^*Min*/+^	(SWR × B6) F1	2	Dove
*Apc*^*Min*/+^	(BR × B6) F1	2	Dove
*Apc*^*Min*/+^	C57BL/6	5	Groden
*Smad3*^-/-^	129	6	Graff
*Smad3*^-/-^	129	7	Coffey
*Tgfb1*^-/-^; *Rag2*^-/-^	C57BL/6	3	Doetschman

Tumors from four established mouse models of CRC (*Apc*^*Min*/+^, AOM, *Smad3*^-/- ^and *Tgfb1*^-/-^*; Rag2*^-/- ^were analyzed. The table provides details on the mouse strains used for the four models, as well as information on the number of samples generated per model and sample-originating laboratory.

#### Human samples: collection/biopsies, regulatory aspects, compliance and informed consents

Sample collection protocol and analyses at the H Lee Moffitt Cancer Center and Research Institute have been described previously [37]. Information collected with the samples for this study includes solid tumor staging criteria for tumor, nodes, and metastases (TNM), Dukes staging/presentation criteria, pathological diagnosis, and differentiation criteria.

#### RNA isolation

All RNA samples were purified using Trizol Reagent from finely dissected tumors and were subjected to quality control screening using the Agilent BioAnalyzer 2100 (Agilent Technologies, Santa Clara, CA, USA).

### Microarray procedures and data analysis

#### Mouse cDNA arrays

Mouse tumors were analyzed on Vanderbilt University Microarray Core (VUMC)-printed 20 K mouse cDNA arrays, composed principally of PCR products derived from three sources: the 15 K National Institute of Aging mouse cDNA library; the Research Genetics mouse 5 K set; and an additional set of cDNAs mapped to RefSeq transcripts. Labeling, hybridization, scanning, and quantitative evaluation of these two-color channel arrays were performed according to VUMC protocols [60] using a whole mouse Universal Reference standard (E17.5 whole fetal mouse RNA). Arrays were analyzed by GenePix version 3.0 (MDS Inc., Sunnyvale, CA, USA), flagged and filtered for unreliable measurements, with dye channel ratios corrected using Lowess and dye-specific correction normalization as previously described [15].

#### Human Affymetrix oligonucleotide arrays

Human RNA samples were labeled for hybridization to Affymetrix HG-U133plus2 microarrays using the Affymetrix-recommended standard labeling protocol (Small-scale labeling protocol version 2.0 with 0.5 μg of total RNA; Affymetrix Technical Bulletin). Microarrays were scanned with MicroarraySuite version 5.0 to generate 'CEL' files that were processed using the RMA algorithm as implemented by Bioconductor [15].

#### Analysis strategy

The four different mouse models of CRC were compared for model-specific differences, then compared to mouse colon development stages, and then to human CRC samples (Figure 1). The mouse tumor sample array data are composed of Lowess-normalized Cy3:Cy5 labeling ratios of each individual tumor sample versus a universal E17.5 whole fetal mouse reference RNA (described using MIAME guidelines in the NCBI GEO database under series accession number GSE5261). The first approach to referencing was to compare normalized ratios across the tumor series. To do this, for each gene, the Lowess-corrected ratio for each probe element (sample versus E17.5 whole fetal mouse reference) was divided by the median ratio for that probe across the entire tumor sample series. This is termed the median-per-tumor expression ratio and was useful for identifying, clustering and visualizing differences that occur between the different tumor samples. Since we previously collected mouse expression data for normal E13.5-E18.5 colon samples from inbred C57BL/6J and outbred CD-1 mice [15] using the identical E17.5 whole fetal mouse reference, this allowed us to combine the data directly. Differential expression profiles in the tumors were combined with relative developmental gene expression levels by direct comparisons of ratios determined within each experimental series. Initial comparisons were made between median normalized tumor data to gene expression levels observed in the E13.5-E18.5 and adult (eight week post-natal) colon samples, which were referenced to either E13.5 samples or to the adult colon. The latter approach subsequently allowed for the broadest comparison of mouse and human data using gene ortholog mapping. Correlated phenomena could be observed from any of the different referencing strategies.

#### Inter-organism gene ortholog and inter-platform comparison strategy

Pairs of human and mouse ortholog genes (12,693) were curated using the Mouse Genome Informatics (MGI; The Jackson Laboratory) [61] and National Center for Biotechnology Information (NCBI) Homologene [62] databases. Individual microarray elements or features were mapped to these. The concatenated human and mouse RefSeq IDs was used as the composite ID for the orthologous gene pair in the ortholog genome definition. NIA/Research Genetics mouse cDNAs were mapped to human orthologs using a variety of resources, usually via the Stanford Online Universal Reference resource [63]. Gene transcript assignments were made unique by choosing the longest corresponding transcript. To map the Affymetrix human and mouse array data into the ortholog genome, we used a sequence matching approach. First, we obtained human and mouse transcript sequences from RefSeq [64] and probe sequences from the manufacturer's website [65]. Next, we computed all perfect probe-transcript pairs. We excluded probes that matched multiple gene symbols but accepted probes that matched multiple transcripts. Probe sets were assigned to represent a given transcript if at least 50% of the perfect match probes of the probe set matched to that transcript. The newly assigned transcript identifiers were then used to map probe sets to ortholog genes. Since some transcripts have multiple probe-set representations on both the Affymetrix and cDNA microarrays to one ortholog identifier, we employed an *ad hoc *strategy to use the average of those probe sets or cDNAs that exhibited consistent regulation across a sample series. In such cases, the signals of the regulated probe sets that were interpreted as being in agreement were averaged and assigned to the corresponding ortholog. We excluded probe sets or cDNAs that we were aware corresponded to non-transcript genomic sequence as tested using BLAT at the UCSC Goldenpath website [66].

Mouse-human RefSeq gene ortholog assignments can be found at GenomeTrafac [67,68]. All ortholog assignments and cross-species mapping annotations were incorporated into annotations associated with the Affymetrix HG-U133 plus2.0 genome. Gene expression ratios obtained for the mouse samples were then represented as expression values within the human platform for all of the probe sets that mapped to the corresponding mouse gene ortholog. Data for the primary human sample series, as well as the combined mouse-human data sets, are available in the Cincinnati Children's Hospital Medical Center microarray data server [69] in the HG-U133 genome under the KaiserEtAl_2006 folders ('guest' login; all cross-platform ortholog gene identifiers are contained as annotation fields within the HG-U133 genome table).

#### Statistical and data visualization approaches

Most normalization, expression-level referencing, statistical comparisons, and data visualization were performed using GeneSpring v7.0 (Silicon Genetics-Agilent (part of Agilent Technologies). Fisher's exact test was performed online at the MATFORSK Fisher's Exact Test server [70]. To identify differentially expressed features between two or more classes, we applied GeneSpring's Wilcoxon-Mann-Whitney or the Kruskal-Wallis test, respectively. For three or more classes, the initial non-parametric test was followed by the Student-Newman-Keuls *post-hoc *test. Results from the primary analyses were corrected for multiple testing effects by applying Benjamini and Hochberg false discovery rate (FDR) correction [71]. In general, due to the referencing strategies, good platform technical performances, and moderately low within-group biological variation of gene expression, stringent cutoffs could be used, that is, the FDR level of significance was set between FDR < 5.10^-5 ^and FDR < 5.10^-4^. K-means clustering was performed using the GeneSpring K-means tool and the Pearson correlation similarity measure.

### Ontology-based analysis of gene cluster-associated functional correlates

Gene expression clusters were analyzed for the occurrence of multiple genes involved in related gene function categories by comparing each list of coordinately regulated clustered genes to categories within Gene Ontology, pathways, or literature-based gene associations using GATACA [72], Ontoexpress [73], and Ingenuity Pathway Analysis, version 3 (IPA, Ingenuity Systems, Redwood City, CA, USA) [74]. To do this, each cluster indicated in Figures 2, 4, 5, 6 and 8 was converted to a list of gene identifiers, uploaded to the application, and examined for over-representation of multiple genes from one or more molecular networks, or functional or disease associations as developed from literature mining. Networks of these focus genes were algorithmically generated based on the relationships of individual genes as derived from literature review and used to identify the biological functions and/or associated pathological processes most significant for each gene cluster. Fisher's exact test was used to calculate a *p *value estimating the probability that a particular functional classification or category of genes is associated with a particular pattern or cluster of gene expression more than would be expected by chance. For each cluster, only the top significant functional classes and canonical pathways are shown. Figure 7a shows a diagram of the canonical WNT signaling pathway and an associated-gene network that was a top-ranked association of the clusters that exhibited significant over-expression in AOM and *Apc*^*Min*/+ ^versus *Smad3*^-/- ^and *Tgfb1*^-/-^*; Rag2*^-/- ^mouse models. Genes or gene products are represented as nodes, and biological relationships between nodes are represented as edges (lines). All edges are supported by at least one literature reference from a manuscript, or from canonical information stored in the Ingenuity Pathways Knowledge Base.

### qRT-PCR

To confirm the validity of data normalization and referencing procedures as well as the cDNA gene assignments of the printed arrays used in the microarray analyses, we used qRT-PCR to measure relative levels of nine genes found by microarray data analysis to be differentially expressed (FDR < 5.10^-5^) in tumors from *Apc*^*Min*/+ ^and *Smad3*^-/- ^mice. Total RNAs from C57BL6 *Apc*^*Min*/+ ^and 129 *Smad3*^-/- ^tumor samples (20 μg) were reverse-transcribed to cDNA using the High Capacity cDNA Archive Kit (oligo-dT primed; Applied Biosystems, Foster City, CA, USA). qRT-PCR reactions (20 μl) were set up in 96-well MicroAmp Reaction Plates (Applied Biosystems) using 10 ng of cDNA template in Taqman Universal PCR Master Mix and 6-FAM-labeled Assays-on-Demand primer-probe sets (Applied Biosystems). Reactions were run on an MX3000P (Stratagene, a division of Agilent Technologies) with integrated analysis software. Threshold cycle numbers (Ct) were determined for each target gene using an algorithm that assigns a fluorescence baseline based on measurements prior to exponential amplification. Relative gene expression levels were calculated using the ΔΔCt method [75], with the *Gusb *gene as a control. Fold-change was determined relative to expression in normal adult colon from two C57BL/6J mice.

### Immunohistochemistry

Immunohistochemical procedures were performed as described [15]. *Apc*^*Min*/+ ^and *Smad3*^-/- ^colon tumors were rapidly dissected, fixed in 4% paraformaldehyde, and embedded in paraffin before cutting 10 μm thick sections. Antigen retrieval was performed by boiling for 20 minutes in citrate buffer, pH 6.0. Sections were treated with 0.3% hydrogen peroxide in PBS for 30 minutes, washed in PBS, blocked in PBS plus 3% goat serum and 0.1% Triton X-100, and then incubated with primary antibodies and HRP-conjugated goat anti-rabbit secondary antibody (Sigma, St Louis, MO, USA). Antigen-antibody complexes were detected with a DAB peroxidase substrate kit (Vector Laboratories, Burlingame, CA, USA) according to the manufacturer's protocol.
